# Determination of Mould and Aflatoxin Contamination in Tarhana, a Turkish Fermented Food

**DOI:** 10.1100/2012/218679

**Published:** 2012-04-24

**Authors:** Hilal Colak, Hamparsun Hampikyan, Enver Baris Bingol, Omer Cetin, Meryem Akhan, Sumeyre Ipek Turgay

**Affiliations:** ^1^Department of Food Hygiene and Technology, Faculty of Veterinary Medicine, Istanbul University, Avcilar, 34320 Istanbul, Turkey; ^2^The School of Vocational Studies, Beykent University, Buyukcekmece, 34500 Istanbul, Turkey; ^3^Academic Hygiene KGaA, Training, Audit and Consulting Services, Kuştepe Mahallesi, Tomurcuk Sokak, İzmen Sitesi, Sisli, 34387 Istanbul, Turkey

## Abstract

Tarhana is a popular traditional Turkish cereal-based fermented food product mainly produced at home or at home-scale level. Some certain mould species can grow even at low moisture and pH values and produce aflatoxins in food. This study was conducted to determine aflatoksin levels in tarhana. For this purpose, a total of 138 tarhana powder samples were collected from bazaars in Istanbul and analyzed for aflatoxins, mould contamination, and some physco-chemical parameters. As a result, 32 out of 138 tarhana samples (23.2%) were found to be contaminated with aflatoxins in the range of 0.7–16.8 *μ*g/kg, whereas 29 samples contained Aflatoxin B1 (AFB1) ranging from 0.2–13.2 *μ*g/kg. All samples (100%) contaminated with moulds in the range of 1.4 × 10^1^ –5.8 × 10^7^cfu/g. The average pH, moisture and a_w_ results were detected as 3.82, 12.71%, and 0.695, respectively.

## 1. Introduction

**Figure 1:**
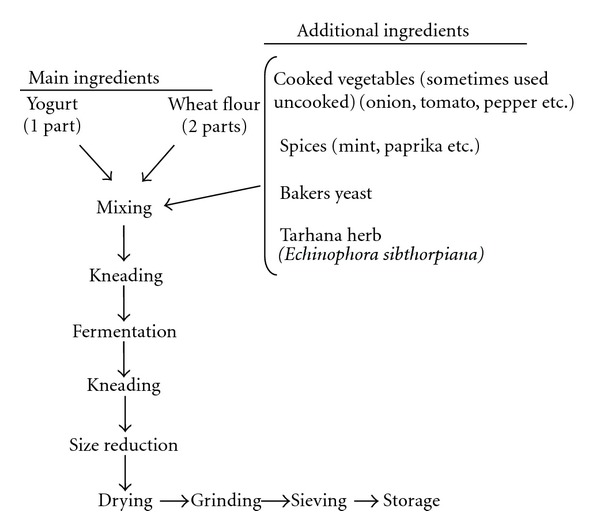
Flow diagram for traditional tarhana production [12].

Fermented cereal-yoghurt mixtures play an important role in the diets of many people in the Middle East, Asia, Africa, and some parts of Europe [1–3]. Safety and some nutritional benefits such as improvement of protein digestibility, degradation of antinutritional factors have been attributed to fermented foods, and therefore they have promoted for safety and nutritional purpose [4]. Tarhana is a popular traditional Turkish cereal-based fermented food product mainly produced at home or at home-scale level [5, 6]. Tarhana is prepared by mixing wheat flour, yoghurt, yeast, salt, some raw or cooked vegetables (tomato, pepper, and onion), and spices (mint, basil, dill, paprika, tarhana herb, etc.) followed by lactic and alcoholic fermentation for one to seven days. The dough at fermentation is called as wet tarhana. After fermentation, the mixture is dried in the sun as a lump, nugget, or thin layers to obtain dry tarhana. Finally, it is ground to powders smaller than 1 mm [1, 4, 5, 7]. Since there is no standard procedure in the production method of tarhana, its nutritional properties depend heavily on the ingredients and the amount used in the recipe [4, 8]. Methods for production of tarhana may vary from one place to another, but cereals and yoghurt are always the major component [2, 3, 9, 10]. Production process of traditional tarhana is shown in Figure 1.

Tarhana is mainly used in the form of a thick and creamy soup reconstituting with water followed by simmering and is consumed at lunch or dinner especially on cold days in Turkey [9, 11]. It is also locally consumed as a snack after it has been dried as thin layer or nugget, not to be ground [8]. There are four different types of tarhana, stated by Turkish Standardization Institute: flour tarhana, göce (cracked wheat) tarhana, semolina tarhana, and mixed tarhana. The difference between them is the usage of the wheat flour, cracked wheat, and semolina separately or as combinations in the recipe [5]. 

Tarhana-like products are known under different names in the other countries: kishk (sour milk-wheat mixture with boiled chicken stock) in Egypt, Syria, Lebanon, and Jordan, kushuk (milk-sour dough mixture with turnips) in Iraq, and tahonya/talkuna (fermented cereal mixture with vegetables) in Hungary and Finland [1, 2, 5, 10, 12].

 Lactic acid bacteria and yeast are responsible for acid formation during fermentation in Tarhana [1]. The low pH (3.8–4.5) and low moisture content (about 10%) of tarhana provide a bacteriostatic effect against pathogenic and spoilage microorganisms [1, 2, 12]. However, some certain mould species such as *Aspergillus, Penicillium*, and *Fusarium* can grow even at low moisture and pH values and produce mycotoxins in several food commodities [13, 14].

Among all mycotoxins, aflatoxins are a group of highly toxic secondary metabolic products named as aflatoxin B1(AFB1), aflatoxin B2 (AFB2), aflatoxin G1 (AFG1), and aflatoxin G2 (AFG2)[15, 16]. Aflatoxins are carcinogenic, mutagenic, teratogenic, and immunosuppressive to most animal species and humans [17]. AFB1 has the highest potency as a toxin and is classified as group I carcinogen by International Agency for Research on Cancer (IARC)[18]. The order of toxicity, AFB1 > AFG1 > AFB2 > AFG2, indicates that the terminal furan moiety of AFB1 is the critical point for determining the degree of biological activity of this group of mycotoxins [19]. Aflatoxins easily occur in feeds and foods during growth, harvest, or storage [20].

Due to their frequent occurrence and toxicity, guidelines and tolerance levels of aflatoxins have been set in several countries including Turkey. According to the Turkish Food Codex, the maximum residue limits for AFB1 and total aflatoxin in risky foods is 5 and 10 *μ*g/kg, respectively [21].

Although several studies are available for aflatoxin levels in different food types which are consumed in Turkey, there is very little information on the presence of aflatoxins in tarhana. On the other hand, limited studies were conducted on mould contamination of tarhana. Therefore, this study was planned to determine aflatoxin levels and mould contamination in tarhana powder which are consumed to a great extend at Turkish kitchen and to compare the obtained results with maximum aflatoxin tolerance limits accepted by the Turkish Food Codex.

## 2. Materials and Methods

### 2.1. Samples

**Figure 2:**
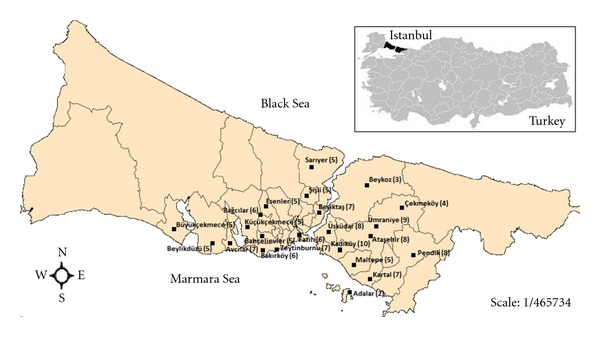
Map of samples collected in Istanbul, Turkey.

During the period September–May 2011, a total of 138 tarhana powder samples were collected randomly from bazaars located in different regions of Istanbul (Figure 2). Samples were transported under cold conditions from their place of collection to the laboratory. The number of samples gathered according to month bought was given in Table 1.

### 2.2. Aflatoxin Analysis

#### 2.2.1. Sample Preparation

Sample preparation procedures were performed according to the instructions of the test kit (Rida Aflatoxin Column Art no.: R5001/5002, R-Biopharm, Darmstadt, Germany) manual [22]. 25 mL of methanol (70%) was added to 5 g of tarhana. Afterwards, the solution was extracted by mixing gently for 10 minutes at room temperature. The extract was filtered through a paper filter and 15 mL of distilled water were added to 5 mL of filtered solution. 0.25 mL Tween 20 were added and stirred for 2 minutes, followed by entire amount of the sample solution (20 mL) passing over the column. Clean up procedure was performed according to the kit's manual. Toxin containing eluate was diluted 1 : 10 with the sample dilution buffer (supplied with the test kit) and used 50 *μ*L per well in the assay.

#### 2.2.2. Test Procedure of Total Aflatoxins

According to Ridascreen Aflatoxin Total (Art no.: 4701) test kit manual [23], 50 *μ*L of the standard solutions or prepared sample in duplicate were added to the wells of microtiter plate. Then 50 *μ*L of the diluted enzyme conjugate and 50 *μ*L of the diluted antibody solution were added to each well. The solution was mixed gently, and incubated for 30 min at room temperature (20–25°C) in the dark. The unbound conjugate was removed during washing for three times (ELISA Washer ELX 50, Bio-tek Inst.). Afterwards, 100 *μ*L of substrate/chromogen solution was added to each well, mixed gently, and incubated for 30 min at room temperature (20–25°C) in the dark. Then, 100 *μ*L of the stop solution (1 M H_2_SO_4_) was added to each well and the absorbance was measured at 450 nm in ELISA plate reader (ELX 800, Bio-tek Inst.). The mean lower detection limit is 0.25 *μ*g/kg.

#### 2.2.3. Test Procedure of AFB1

According to Ridascreen Aflatoxin B1 30/15 (Art no.: 1211) test kit manual [24], 50 *μ*L of the standard solutions or prepared sample in duplicate was added to the wells of microtiter plate. Then 50 *μ*L of the enzyme conjugate and 50 *μ*L of the anti-aflatoxin antibody solution were added to each well, mixed gently and incubated for 30 min at room temperature (20–25°C). The washing procedure was applied for three times (ELISA Washer ELX 50, Bio-tek Inst.). After the washing step, 100 *μ*L of substrate/chromogen solution were added to each well and mixed gently and incubated for 30 min at room temperature (20–25°C) in the dark. Finally, 100 *μ*L of the stop solution (1 M H_2_SO_4_) were added to each well and the absorbance was measured at 450 nm in ELISA plate reader (ELX 800, Bio-tek Inst.). The mean lower detection limit is 1.0 *μ*g/kg.

#### 2.2.4. Determination of Moisture and Water Activity

Moisture contents of tarhana samples were determined by drying a homogeneous mixture of the sample in an oven (Heraeus, Germany) at 105 ± 2°C until a constant weight was obtained according to AOAC procedures [25]. The water activity analysis was determined by means of water activity meter (Decagon, AquaLab Lite, USA).

#### 2.2.5. Determination of pH

The pH was determined after mixing a 10 g sample with 90 mL distilled water (1/10 sample/water) and the pH value measurements were carried out using a Hanna pH meter (Hanna HI-9321, Woonsocket, Rhode Island, USA), equipped with a FC220B electrode (Hanna HI-9321, Woonsocket, Rhode Island, USA), after calibration with standard buffers of pH 4.0 and 7.0 [26].

#### 2.2.6. Mould Analysis

Mould was defined on Dichloran Rose Bengal Chloramphenicol agar with Chloramphenicol Selective supplement (DRBC, Oxoid, CM0727, and SR0078). Spread plates were incubated at 25°C for 5 days [27].

## 3. Results and Discussion

The distribution and evaluation of mould counts, aflatoxin amounts, pH, moisture, and water activity values of analyzed tarhana samples are given in Tables 2, 3, and 4, respectively.

**Figure 3:**
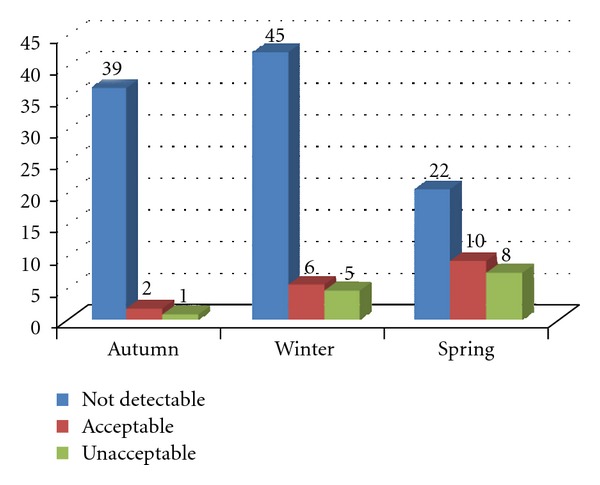
The number of acceptable and unacceptable samples according to Turkish Food Codex (TFC).

In this study 32 (3 in autumn, 11 in winter, 18 in spring) out of 138 tarhana samples (23.2%) were found to be contaminated with aflatoxins in the range of 0.7–16.8 *μ*g/kg, whereas 29 out of 138 (21.0%) tarhana samples contained AFB1 ranging from 0.2–13.2 *μ*g/kg (Table 3). According to these results, 14 tarhana samples exceeded the maximum limits of AFB1 (5 *μ*g/kg) and total aflatoxin (10 *μ*g/kg) set in the Turkish Food Codex [21] (Figure 3). 

Because the presence of aflatoxins in food is a hazard to human health, numerous studies have been conducted in different countries and also in Turkey to examine the presence and levels of aflatoxins in various food commodities. However, there is very little information on the presence of aflatoxins in tarhana, except for a report by Arici [28], who detected AFB1 in 4 out of 31 (12.9%) tarhana samples. Our results were higher than the results of the above-mentioned researcher. 

The mould contamination rate on examined tarhana samples was fairly high. As can be seen from Table 2, all samples (100%) contaminated with moulds in the range of 1.4 × 10^1^ –5.8 × 10^7^ cfu/g. The average count was detected as 4.6 × 10^3^ cfu/g. 

In Turkey, there are few studies on mould contamination in tarhana. In a study conducted by Soyyigit [29], the yeast-mould counts were detected as <10–3.3 × 10^7^ cfu/g in 27 examined tarhana samples produced in Isparta city. Coskun [30] reported the mean yeast-mould counts in tarhana samples as 3.04 × 10^3^, 3.52 × 10^3^, and 3.37 × 10^1^ cfu/g in Edirne, Kırklareli and Tekirdağ cities, respectively. Daglioglu et al. [31] found the mould/yeast contamination at a level of 1.5 × 10^3^ cfu/g in traditional dried tarhana samples. 

As expected, samples with high mould contamination contained high aflatoxin levels (Table 3). Source of mould and aflatoxin contamination in tarhana may result from wheat flour and spices used in the production. Wheat flour is an ingredient used in many foods in European and American culture and also is main ingredient of tarhana. Flour is generally regarded as a microbiologically safe product as it is a low water activity commodity [32]. However, toxigenic moulds may contaminate and grow in flour at different phases of production and processing, mainly in appropriate humidity and temperature conditions. Hence, there are several researches on mould contamination and aflatoxin levels in wheat and wheat flour [32–34]. Spices are exposed to a wide range of microbial contamination due to poor collection conditions, unpretentious production process, and extended drying times. In addition, spices can be contaminated through dust, waste water, and animal/human excreta in unpackaged spices which are sold in markets and bazaars. Several studies have demonstrated that spices are contaminated with various microorganisms including toxigenic moulds (especially *Aspergillus *spp.) and aflatoxins [35]. Therefore, spices pose health problems because they are often added to foods without further processing or are eaten raw. Therefore, to protect the consumer's health, it should not be used mould and aflatoxin-contaminated flour and spices in tarhana production. 

As can be seen from Table 4 the average pH, moisture, and *a*_*w*_ results were 3.82, 12.71%, and 0.695, respectively. Similar results were also reported by other researchers [3, 9, 36, 37]. It is obvious that moisture has a great importance for the safe storage of food regarding microorganisms, particularly certain species of moulds. In addition to this, poor hygienic production conditions and absence of standard production method of tarhana may enhance of aflatoxin production by moulds. 

In conclusion, aflatoxin producing mould species contaminate numerous food commodities, in warm climates where they may produce aflatoxins at different points of the food chain, such as preharvest, processing, transportation, or storage. The results of this study demonstrated that in spite of the low moisture and pH levels, moulds may grow and synthesized aflatoxins in tarhana. In order to prevent the health risk, a number of methods (storing in proper moisture and temperature, standardization of production method, improving the production conditions, microwave treatments, packing, etc.) can be applied to reduce/eliminate moulds from tarhana.

## Figures and Tables

**Table 1:** Number of samples collected according to month.

Season	Month	Year	Number of sample
Autumn	September-October-November	2010	42
Winter	December-January-February	2010-2011	56
Spring	March-April-May	2011	40

**Table 2:** Distribution and evaluation of mould contamination in Tarhana samples (*n* = 138).

Range (cfu/g)	ND* (<10)	10 to <10^2^	10^2^ to <10^3^	10^3^ to <10^4^	10^4^ to <10^5^	10^5^ to <10^6^	10^6^ to <10^7^	>10^7^
Number	—	13	22	43	32	15	8	5
Percentage (%)	—	9.4	15.9	31.2	23.2	10.9	5.8	3.6

**Table 3:** Mould counts and Aflatoxin amounts of contaminated tarhana samples.

Season	Sample no.	Mould Count (cfu/g)	Total aflatoxins ± SE (*μ*g/kg)	AFB1 ± SE (*μ*g/kg)
Autmn	No: 4	3.8 × 10^5^	4.1 ± 0.1	2.9 ± 0.3
	No: 7	1.3 × 10^6^	10.5 ± 0.2	9.7 ± 0.6
	No: 11	4.1 × 10^5^	1.2 ± 0.1	0.7 ± 0.4

Winter	No: 18	2.1 × 10^4^	3.6 ± 0.7	ND
	No: 25	3.5 × 10^6^	15.1 ± 0.8	8.8 ± 0.9
	No: 32	5.7 × 10^6^	16.2 ± 0.9	10.4 ± 0.4
	No: 45	5.8 × 10^7^	16.8 ± 0.5	13.2 ± 0.3
	No: 48	1.9 × 10^5^	8.2 ± 0.6	4.4 ± 0.2
	No: 53	1.2 × 10^5^	6.2 ± 0.9	3.4 ± 0.2
	No: 57	6.9 × 10^5^	11.9 ± 0.3	10.2 ± 0.7
	No: 59	4.3 × 10^4^	3.4 ± 0.1	ND
	No: 63	1.2 × 10^7^	14.1 ± 0.2	10.4 ± 0.7
	No: 68	4.1 × 10^5^	2.6 ± 1.1	2.1 ± 0.3
	No: 71	4.8 × 10^6^	12.1 ± 0.3	7.5 ± 0.4

Spring	No: 74	5.2 × 10^4^	7.6 ± 0.5	4.8 ± 0.8
	No: 76	3.5 × 10^4^	3.1 ± 0.7	2.6 ± 0.4
	No: 80	2.2 × 10^6^	11.6 ± 0.9	6.8 ± 0.7
	No: 82	2.8 × 10^5^	1.7 ± 0.2	0.8 ± 0.3
	No: 87	6.1 × 10^3^	0.9 ± 0.1	0.5 ± 0.1
	No: 95	1.1 × 10^7^	10.8 ± 1.2	8.7 ± 0.4
	No: 99	4.5 × 10^3^	2.7 ± 0.3	1.8 ± 0.2
	No: 101	1.7 × 10^6^	14.3 ± 1.1	7.8 ± 0.9
	No: 107	5.8 × 10^5^	11.1 ± 0.9	8.3 ± 0.6
	No: 108	2.4 × 10^7^	12.5 ± 0.5	9.1 ± 0.3
	No: 111	1.8 × 10^4^	0.7 ± 0.2	0.2 ± 0.1
	No: 113	6.2 × 10^6^	12.3 ± 0.8	10.6 ± 0.4
	No: 115	6.4 × 10^5^	5.2 ± 0.5	3.1 ± 0.2
	No: 119	3.2 × 10^7^	15.1 ± 0.8	8.6 ± 0.5
	No: 120	1.8 × 10^3^	2.9 ± 0.3	1.1 ± 0.2
	No: 125	7.2 × 10^5^	4.5 ± 0.6	1.4 ± 0.5
	No: 127	3.2 × 10^4^	6.2 ± 0.2	3.8 ± 0.7
	No: 134	3.1 × 10^3^	0.8 ± 0.3	ND

ND: not detected, SE: standart error.

**Table 4:** pH, moisture, and water activity results of analyzed tarhana samples.

Parameters	Minimum	Maximum	Average
pH	3.25	4.50	3.82
Moisture (%) (Dry matter %)	10.35 (89.65)	17.85 (82.15)	12.71 (87.29)
Water activity (*a*_*w*_)	0.658	0.895	0.695
